# Precise detection of coal and gangue based on natural γ-ray

**DOI:** 10.1038/s41598-024-51424-w

**Published:** 2024-01-13

**Authors:** Ningbo Zhang, Changyou Liu, Chuanqi Zhu, Baobao Chen, Zhongbin Wang, Xiaojie Wu

**Affiliations:** 1 School of Mines, China University of Mining and Technology, Xuzhou, China; 2 State Key Laboratory of Mining Response and Disaster Prevention and Control in Deep Coal Mines, Anhui University of Science and Technology, Huainan, China; 3 School of Mechatronic Engineering, China University of Mining and Technology, Xuzhou, China; 4 School of Electrical Engineering, China University of Mining and Technology, Xuzhou, China

**Keywords:** Mineralogy, Coal

## Abstract

To address the technical limitations of automatic coal and gangue detection technology in fully mechanized top coal caving mining operations, the low radiation level radioactivity measurement method is utilized to assess the degree of coal-gangue mixture in top coal caving process. This approach is based on the distinguishing radiation characteristics of natural γ-rays between coal and gangue. This study analyzed the distribution characteristics of natural γ-rays in coal and rock layers of thick coal seams and the applicability of this method, introduced the basic principle of coal-gangue detection technology based on natural γ-ray, developed the test system about automatic coal-gangue detection, studied the radiation characteristics of coal and gangue, proposed determination model of the coal-gangue mixed degree, combined with the time sequence characteristics of the top coal’s releasing flow and the energy spectrum characteristics of different layers of rock, realized the precise coal-gangue detection technology in complex structure thick coal seam with multiple gangue. Field tests were conducted in Lilou, Xiaoyu and Tashan Coal Mine. The test results were well corroborated with the research results and achieved the expected results, which laid the foundation for the field application of intelligent coal mining.

## Introduction

Nearly half of China’s coal resource reserves and output are attributed to thick coal seams^1–3^. The fully mechanized longwall top coal caving (LTCC) mining technology is one of the main technologies used for mining thick seams^4–7^. Improper timing of the caving-opening closure in the caving process of the LTCC mining face can result in excessive or insufficient caving, leading to resource wastage or compromising coal quality^8,9^. Reasonable time for closing the caving-opening depends on the mixing degree of coal gangue^10–12^. Therefore, accurate, real-time and effective technology for identifying coal gangue mixing degree is an effective measure to realize automation and intelligent mining in coal mines, as well as promoting the intelligent industrialization of coal industry^13–15^. It can effectively reduce labor cost, enhance the safety of coal mining operations, reduce equipment maintenance expenses, and significantly improve the coal mining extraction rate, ultimately leading to higher productivity and efficiency^7,16,17^.

In recent years, many experts and scholars have conducted extensive scientific research in the field of coal and gangue detection^18^. Qingjun Song et al. proposed various methods to identify the vibration sound signal emitted by hydraulic support tail beam in the process of coal and gangue collapsed, aiming to realize detection of coal and gangue^16,19^. Bingxiang Huang et al. proposed a coal gangue detection method utilizing near-infrared spectroscopy^20^. Jiachen Wang et al. employed the top coal tracker to monitor the movement of top coal and achieve automated coal drawing in combination with coal gangue image recognition. Chuangyou Liu et al. proposed an coal gangue identification method using active microwave irradiation infrared detection. Zengcai Wang et al. proposed γ Detection of coal gangue mixing rate in top coal caving mining by radiographic method^12,17,21,22^. Liansheng Li et al. put forward a method for identifying coal and gangue based on density difference^23^, and Feng Xing proposed a non-contact microwave detection technology to detect the mixing degree of coal and gangue^24^. Jingjing Deng et al. employed terahertz technology to generate images of coal gangue mixture for the purpose of coal gangue detection^25–28^. Yuming Huo optimized the parameters of intelligent coal drawing process by establishing a predictive model of the periodic coal drawing time^29,30^.

The research above on the recognition of coal and gangue in LTCC mining, primarily adopt the principles of image grayscale, sound spectrum, vibration spectrum, natural γ method, and various composite monitoring methods. However, it is evident from the research objects and results that this study is still in the preliminary stage, primarily due to the complex structural characteristics of extremely thick coal seams in China, which often contain multiple layers of dirt bands^31,32^. The presence of dirt bands results in a mixed flow containing top coal, dirt bands and roof rocks flowing out of the top-coal caving-opening during the top coal caving process of the working face^33^.

That is, the coal gangue detection method in LTCC mining should not only address the detection of coal gangue mixing ratio in coal seams with a simple structure, but also accommodate complex structure coal seams^32,34^. In order to achieve the objective of real-time and accurate recognition of the mixing degree between coal and gangue in LTCC mining, this paper proposes an accurate recognition method based on natural γ ray, which utilizes the radiation difference characteristics of coal and rock natural γ ray. A low radiation level radioactive measurement method is employed to determine the instantaneous mixing ratio of coal and gangue mixture during the top coal caving process, thereby laying the foundation for realizing the intellectualization of LTCC mining.

## Methods

### Distribution characteristics of natural radionuclides in thick coal seams

#### Natural radionuclide

Natural radionuclides are formed during interstellar processes, such as the Big Bang, and continue to exist. They were transported to Earth during its formation. Currently, there are three natural radioactive series (uranium, actinium and thorium) and some non-series radionuclides in nature, however, only the former three can significantly impact radiometric measurement, as illustrated in Table 1.

**Table 1:** The characteristics of three kinds of natural radioactive substances.

Nuclide	Number of nuclide decays/g s	Number of photons produced by each decay of nuclide	Average photon energy/MeV
U	1.23 × 10^4^	2.24	0.80
Th	4.02 × 10^3^	2.51	0.93
K	31.3	0.11	1.46

### Deposition characteristics of natural radionuclides in coal beds

Natural radionuclides are present in various types of rocks, including coal-bearing strata^35–37^. In LTCC mining, the natural ray coal gangue identification technology heavily relies on the immediate roof of the coal seam. As such, the roof deposition characteristics of thick and extra-thick coal seams were studied, and the characteristics of their natural radiation intensity were analyzed.

Factors influencing the content and distribution of radionuclides in sedimentary rocks comprise the sediment source, composition and structure of the rocks, sedimentary conditions and sedimentary environment, radionuclide content of parent rock, duration of radionuclide presence, sediment grain size, and distance from the original location. Thus, the abundance of radionuclides in rocks is influenced by factors related to their formation mode, location and temporal aspects. For natural rocks, the distribution of radionuclides is as follows:

1. Rocks or minerals of a similar nature exhibit comparable levels of radionuclide abundances.
2. There are significant variations in the abundance of radionuclides among different rocks or minerals.

These aforementioned laws possess statistical characteristics and exist objectively, forming the basis for the coal gangue identification technology by natural gamma-ray.

To sum up, the coal mine roof exhibits varying radiation characteristics due to the diverse composition of sedimentary rocks, resulting in significant variations in the levels of uranium, thorium and potassium.

The radionuclides content in the roof primarily correlates with the grain size of the sediment, the amount of organic substances in the sedimentary environment, the sedimentary environment and conditions, the sedimentary time and other factors. Consequently, the following general rules apply:

1. The content of radionuclides in rocks of the same type is similar. The content of radionuclides in different rocks varies greatly.
2. In the coal bearing rock series, the lowest radioactive intensity is coal, while the radioactivity of conglomerate, coarse sandstone, medium sandstone, fine sandstone, siltstone, sandy mudstone, shale and mudstone gradually increases. The smaller the particle size of diagenetic material, the greater the mud content and the stronger the radiation.
3. Inland roof rock exhibit lesser radiation compared to offshore roof rock. The presence of asphaltene mudstone, phosphorite and organic matter in the offshore sedimentary rocks contributes to the effective absorption of radionuclides during sedimentation, resulting in a generally higher radiation level compared to inland type rocks.
4. The shorter coal forming time generally means stronger roof radiation. Thick coal seams are mostly lignite with low metamorphic degree, and their roof formation time is less than bituminous coal and anthracite, so their roof radioactivity is relatively large. Therefore, for similar roof rocks, the shorter formation time means the greater radiation intensity, which is beneficial to the application of natural ray coal gangue detection technology.
5. Sedimentary rocks containing diagenetic minerals such as potash exhibit high radiation content. In order to identify the source of natural radiation accurately, it is necessary to analyze the composition of diagenetic minerals during measuring the radiation of coal mine roof.

**Figure 1:**
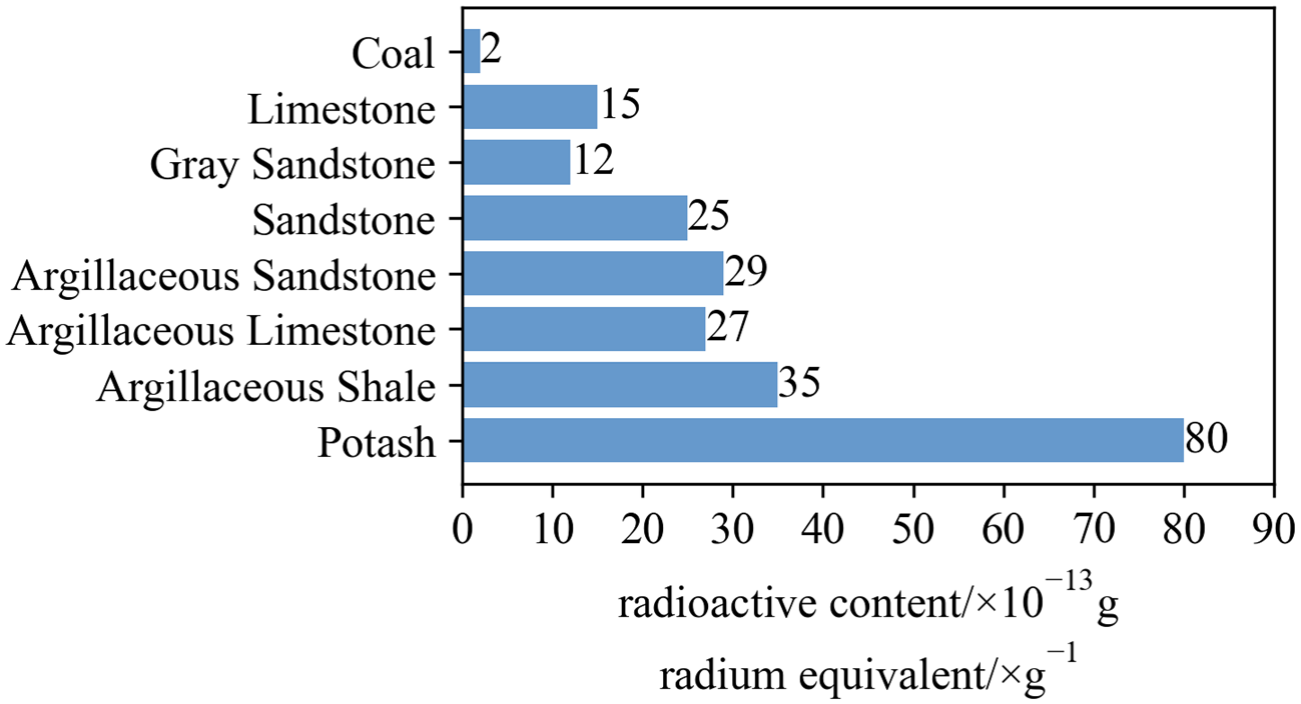
Comparison chart of sedimentary radioactive content.

As depicted in Fig. 1 that the radioactivity content of coal is the lowest. If the shielding effect of loose coal is considered, the radioactivity of coal can be ignored. The radioactivity of potassium salt is the highest. The radioactivity of common roof rocks in coal mines such as sandstone is more than 5 times that of coal. It is with a large different, therefore, it is feasible to obtain the content of gangue in the mixture by measuring the radiation intensity in the mixture of coal and gangue.

### Characteristics of roof rock property of LTCC face in extra thick coal seam in China

**Figure 2:**
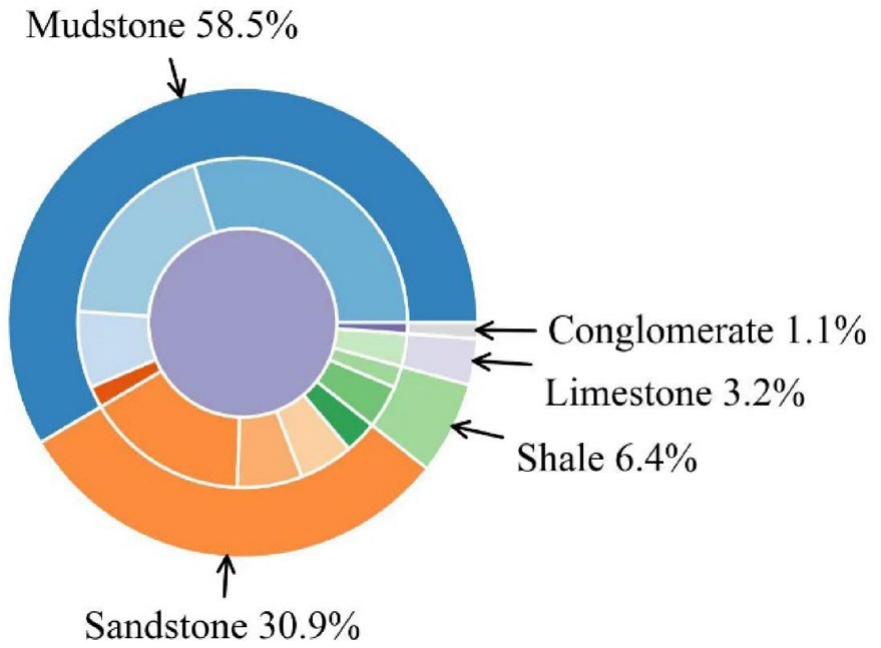
Immediate roof lithologic distribution of full-mechanized caving mining face.

According to the statistics of China National Knowledge Infrastructure Net documents, the immediate roof lithology of 94 LTCC faces in China is shown in Fig. 2.

As depicted in Fig. 2, among the 90 working faces surveyed, mudstone is the most prevalent roof rock type, representing 58.5% of the total statistical data, followed by sandstone at 30.9%, while conglomerate and limestone hav substantially lower occurrences. Among the sandstones, fine grained siltstone represented the primary type of sanedstone, accounting for 51.7% of the sandstone roof.

According to Fig. 1, about 86% thick coal seams in China have significant differences in immediate roof radiation intensity from coal seams, so natural γ X-ray technology has wide applicability in coal gangue identification.

### Radiation characteristics of coal and rock strata in typical thick coal seam mining areas in China

It can be seen from the above analysis that the radionuclide content of sedimentary rocks is related to the content of clay minerals, formation time, environment of sedimentary area and other factors. Therefore, representative typical mining areas such as Dongsheng, Datong, Yanzhou, Shuozhou and Longkou are selected to analyze and study the radiation characteristics of coal and roof rock in thick and extra thick coal seams.

**Figure 3:**
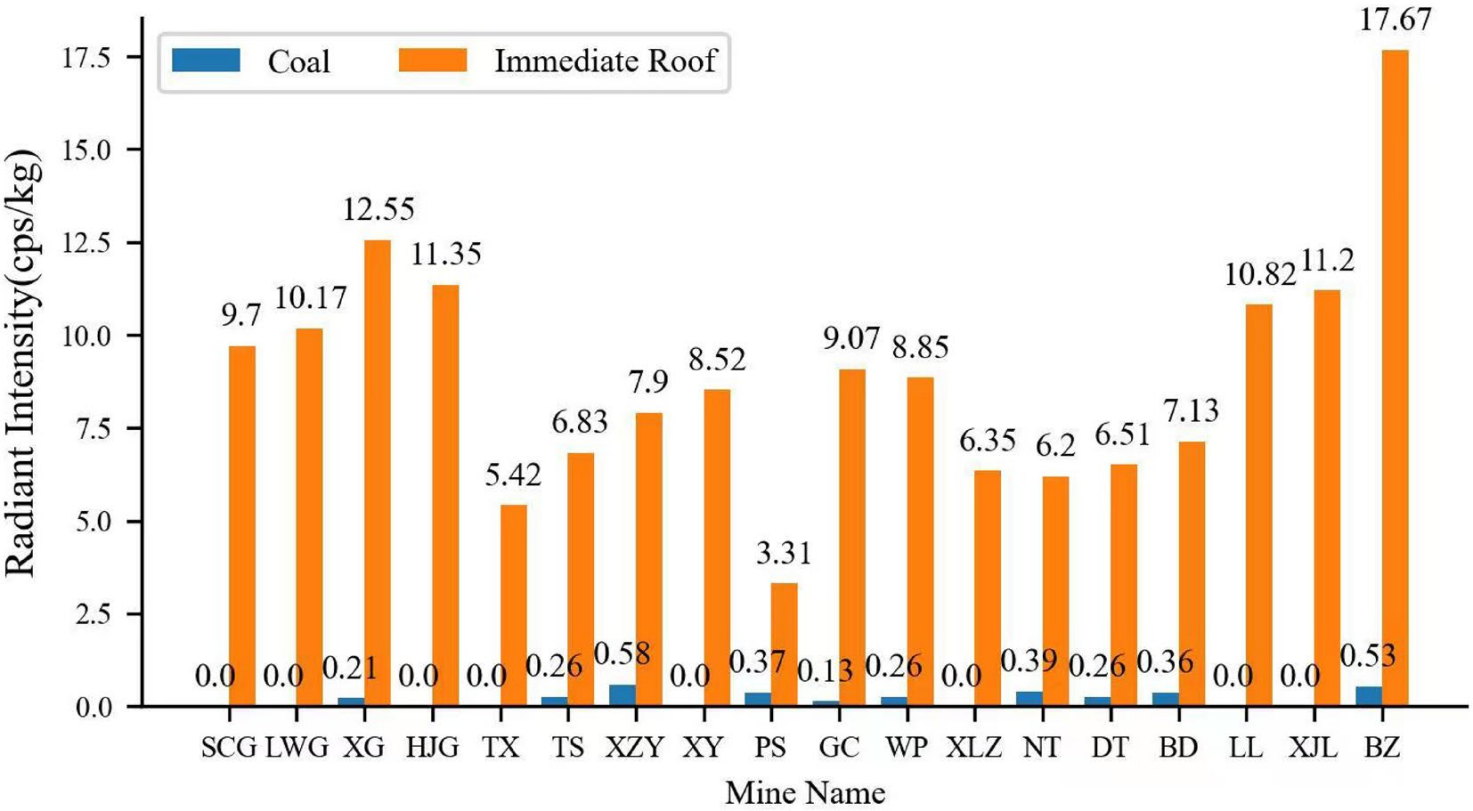
Radiation characteristics of typical thick coal measures.

As depicted in Fig. 3, ① there are radioactive elements in coal and rock, and the radiation intensity of coal samples is generally small, even less than their own shielding capacity. Therefore, in this paper, the radiation content of coal is considered as 0. The radiation intensity of roof rock is much higher than that of coal sample, and the difference can be several times or even dozens of times. Therefore, the mixing degree of coal and gangue can be identified through the difference of radiation characteristics between coal and rock; ② The difference of radiation between different rocks is huge, so it can be realized to distinguish and identify the dirt band and roof rock in the complex structure coal seam through the difference of radiation characteristics between different rocks; ③ The radiation intensity of rock samples from the same sedimentary rock stratum in the same coal field is similar, so for the same working face or even the same coal seam, there is no need to frequently adjust the parameters in the process of using the ray coal gangue identification technology.

### Basic principle of natural γ-ray coal and gangue recognition

The principle of natural γ-rays coal and gangue recognition is summarized as follows:

Based on the radiation differentiation characteristics of natural γ-rays in coal and rock, the method of low radiation level radioactivity measurement is adopted to identify the instantaneous mixing rate of coal and gangue flow in coal releasing process. Combined with the time-series characteristics of caving flow of top coal in fully mechanized caving mining and the energy spectrum characteristics of different strata, the automatic identification of coal and gangue in fully mechanized caving of thick coal seam with complex structure containing multiple gangue is realized.

1. In the process of top coal caving, the gangue outflow from the caving-opening has a changing law from nothing to existence, from less to more.
2. In the process of top coal caving, the natural radiation intensity in the coal and gangue mixture changes from weak to strong, and the content of gangue in the mixed flow can be determined.
3. By detecting the instantaneous radiation intensity of the coal and gangue mixed flow to determine the rate of gangue and thus determine the time to close the coal drain.
4. For coal beds with complex structure containing one to multiple layers of gangue, the influence of gangue inclusion on the accuracy of coal and gangue recognition can be excluded according to the different energy spectrum characteristics of gangue inclusion and the caving time sequence characteristics, which is, the coal and waste collection at different levels will be caved in the sequence of time according to the different positions from the caving-opening in the process of caving.

**Figure 4:**
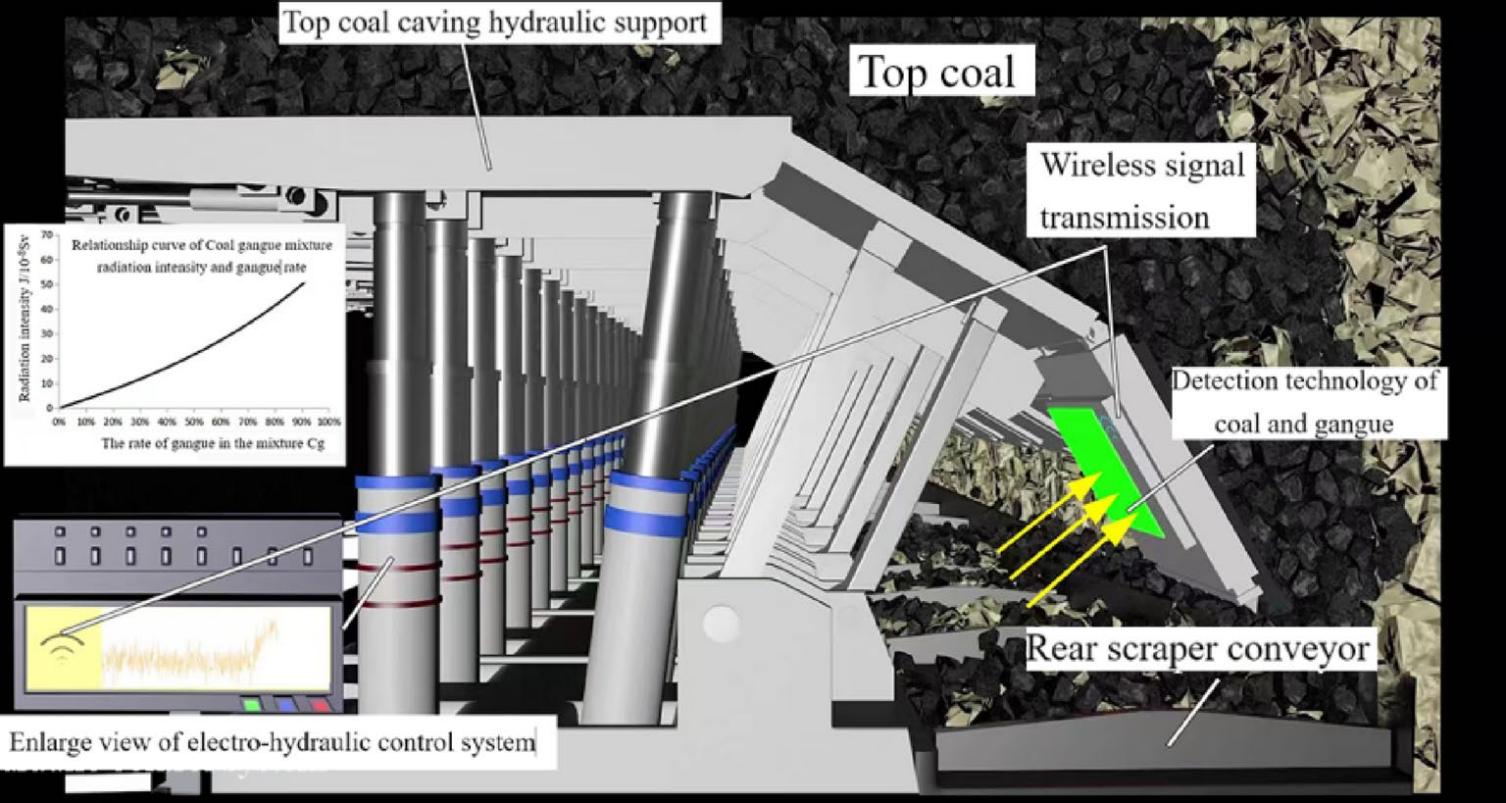
Schematic diagram of natural γ-ray coal and gangue identification.

The schematic diagram of natural γ-ray coal and gangue recognition is shown in Fig. 4.

### Development of coal and gangue identification system

The developed mine intrinsic safe coal and gangue natural ray real-time detection system includes two parts: detector and data acquisition display terminal. The detector is composed of NaI crystal(Ф100 × 100), photomultipler, shell, data processing terminal and explosion-proof shell. The data processing terminals include coal and gangue identification system APP and intrinsic safe Android mobile phones. The data acquisition display terminal and the detector will be connected wirelessly for data transmission. During the measurement at the LTCC face under the shaft, the single chip microcomputer will be used for data analysis, processing and display. The system and related indicators are shown in Table 2.

**Table 2:** Technical index of coal and gangue identification system.

Detector range	Background number	Sensitivity	Energy threshold	Relative error	Minimum sampling period
0.001–100uGy/h	≥ 100cps	1uGy/h ≥ 1000cps	35 keV	≤ ± 5%	50 ms

Due to the difference between the background radiation of the underground environment and that of the laboratory environment, parameter debugging is required before the dynamic monitoring of the coal drawing mouth to adapt to the underground radiation environment.

**Figure 5:**
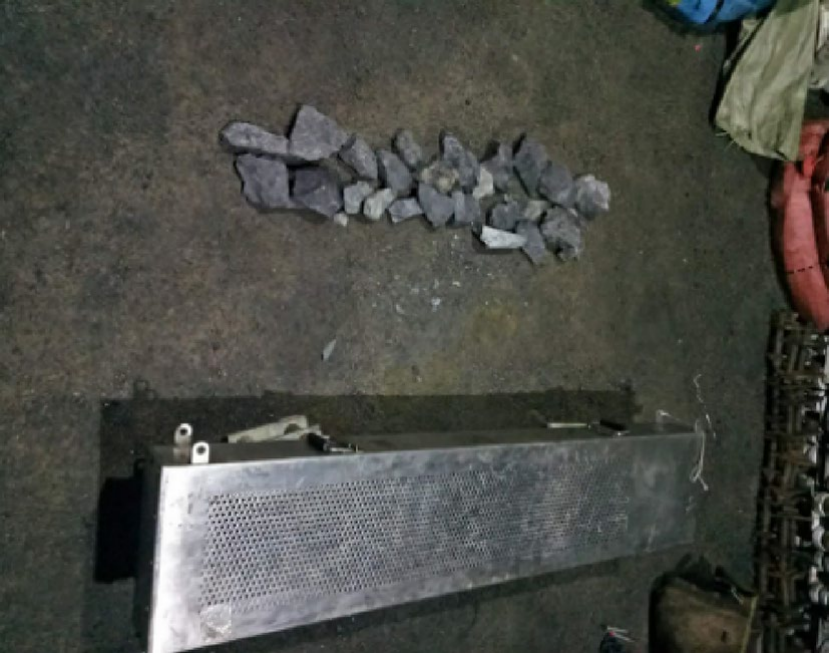
Detector parameters adjustment chamber placement.

The detector is placed in the material chamber of 1303 working face. The chamber floor is a coal seam covered with sand and gravel. Adjust the detector detection face upward and downward once, and the bottom shall be padded 20 cm above the ground, as shown in Fig. 5.

(1) Influence of supply voltage.

**Figure 6:**
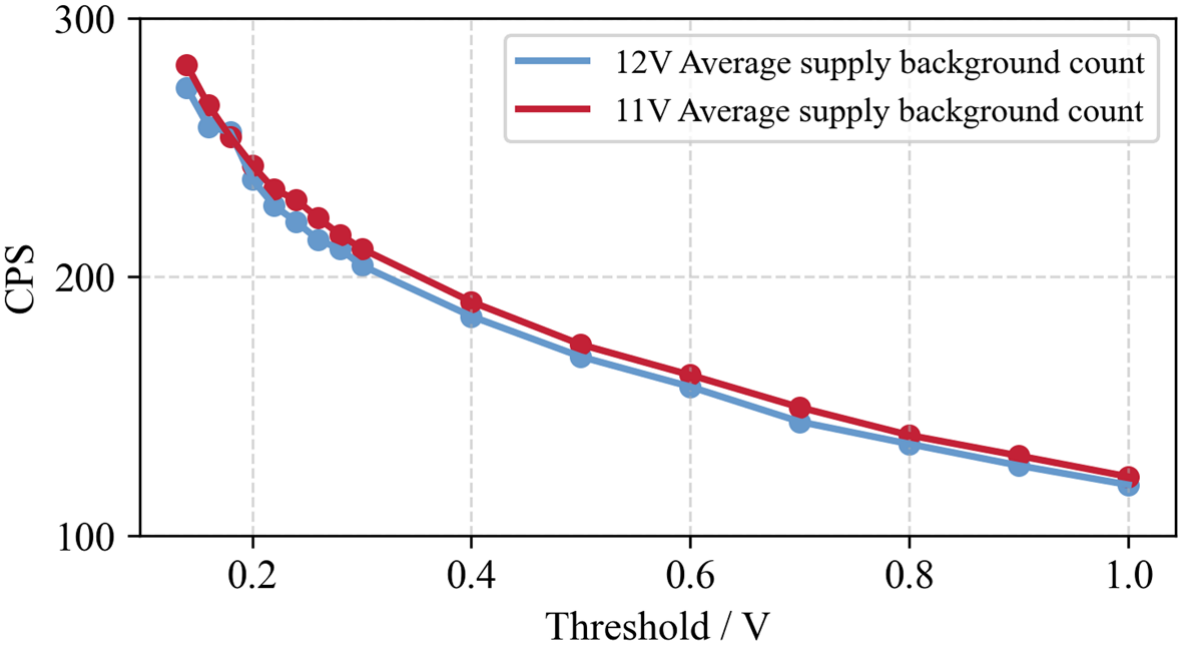
Comparison of detector counting under different supply voltages.

In order to test the influence of different power supply voltages of the circuit on the detection efficiency of the detector, adjust the counting conditions when the power supply voltage of the detector is 11 V and 12 V, as shown in Fig. 6.

As depicted in Fig. 6 that under the situation that other conditions remain unchanged and only the power supply voltage is changed, the count value of the detector is relatively stable, which can verify that the circuit can work normally under the power supply condition of more than 11 v.

(2) Threshold debugging.

**Figure 7:**
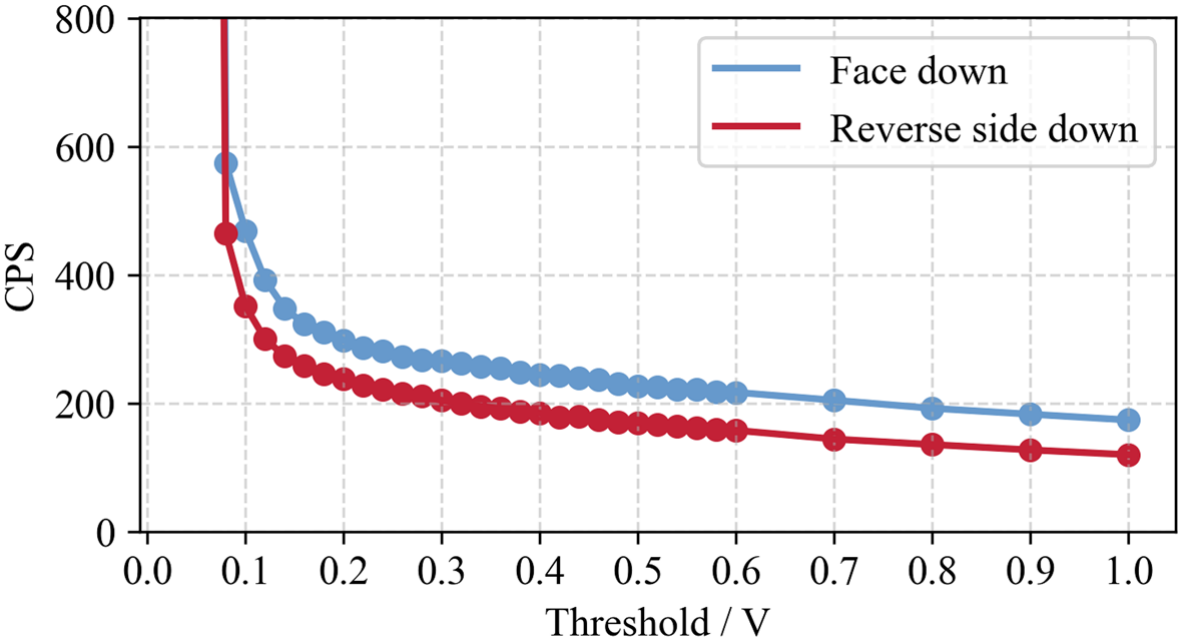
Detector background CPS values under different thresholds.

The threshold adjustment range is 0–1 v, and the adjustment amplitude is 0.02 v. The test results are shown in Fig. 7.

As depicted in Fig. 7, (1) with the increase of threshold value, the counting value of the detector shows a downward trend; (2) under the threshold value of 0–0.08 v, the counting of the detector is too large, and after exceeding 0.08 v, the counting of the detector is in the normal range; (3) the detection surface of the detector is directional, that is, the counting of the detector is not only related to the position of the detector, but also related to the direction of the detection surface.

(3) Background comparison between ground and underground environments.

**Figure 8:**
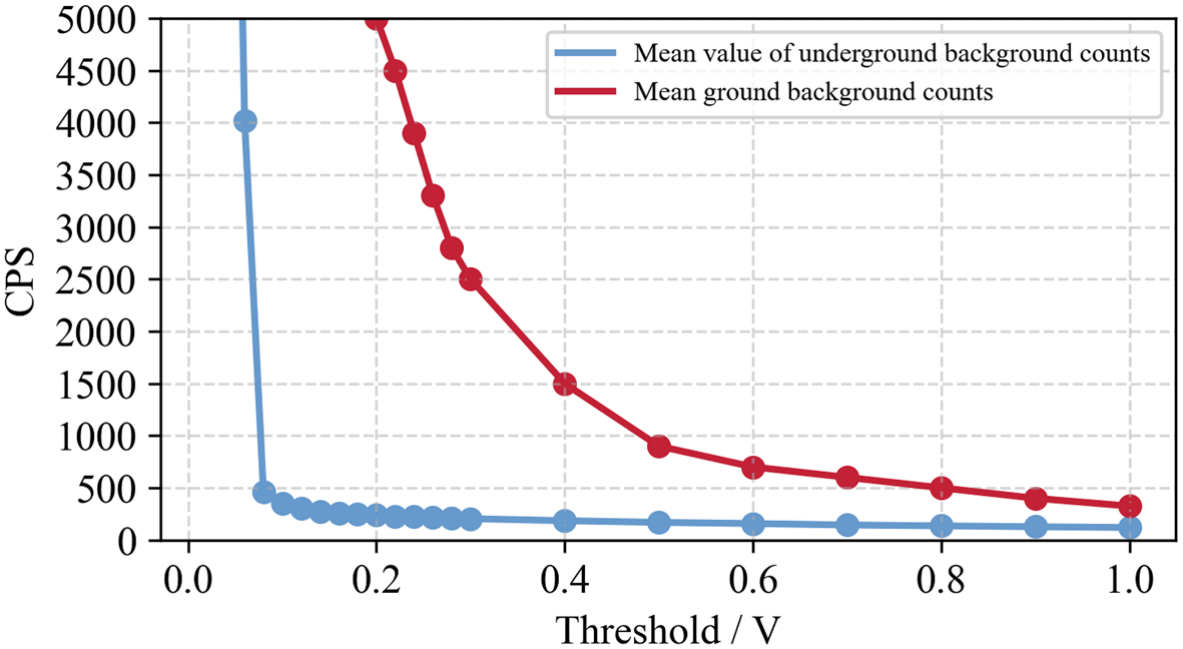
Radiation difference under different environmental conditions above and below surface.

In order to compare the radiation difference under different environmental conditions above and below the surface, the background counts of the detector are counted at different thresholds, as shown in Fig. 8.

**Figure 9:**
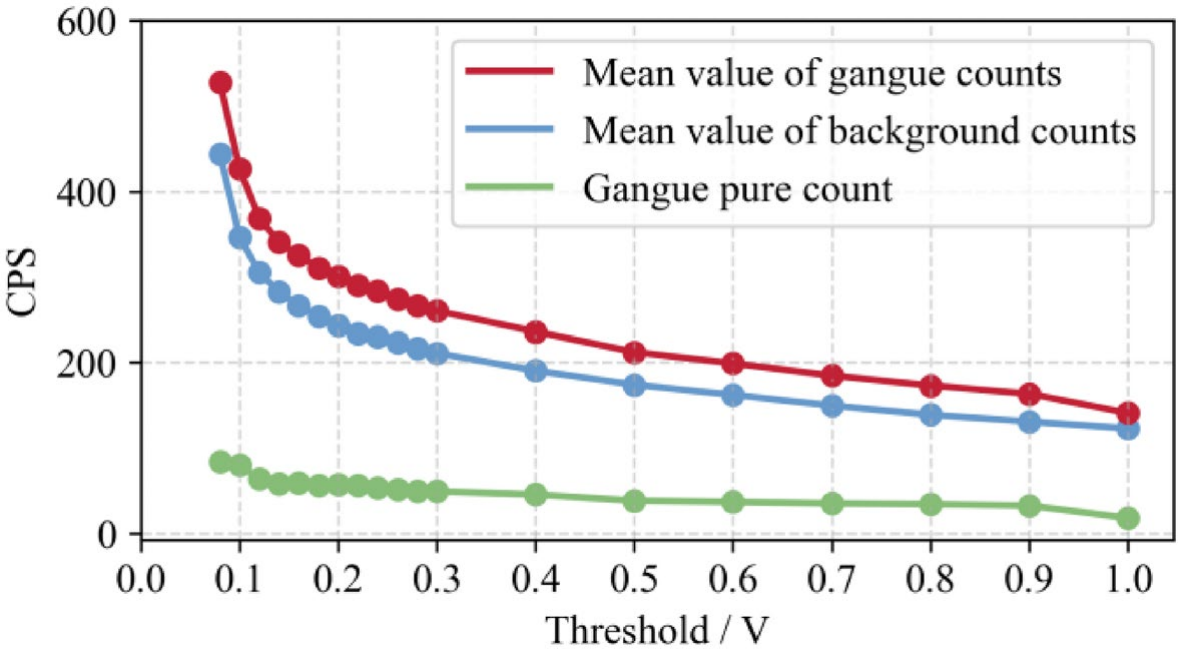
Radiation intensity test of gangue in coal mine environment.

As depicted in Fig. 9 that after exceeding 0.08 v, the underground environment detector counts in the normal range, and the technology slowly decreases with the increase of threshold; In the surface environment, the count is in the normal range only when the threshold value is 0.5 v. This shows that the radiation field of the ground environment is far more complex than that of the underground environment. As the radiation content of rock is not affected by the change of detection location, the comparison between the presence of gangue and the absence of gangue will be more obvious during underground detection than that on the ground.

In order to verify the detection effect of gangue radiation in the underground environment, the static test is carried out in the material roadway chamber of the underground working face. Put the detector in the chamber, with the detection face upward and the bottom pad 20 cm above the ground.

During the test, first count the background under different threshold values (0.08–1 V), then place gangue (about 10 kg), and count again under different threshold values (0.08–1 V). Figure 9 is the specific data.

As depicted in Fig. 9 that the radiation intensity of the same pile of gangue decreases with the increase of the threshold value, and the background value also descends. Because the background radiation of the underground environment comes from the radioactive elements in the roof and floor rocks and the radioactive elements in the air, of which the radioactive elements in the roof and floor rocks account for the majority. Therefore, the energy spectrum of the background is close to that of the gangue, and the adjustment of the threshold value will affect the counts of both.

**Figure 10:**
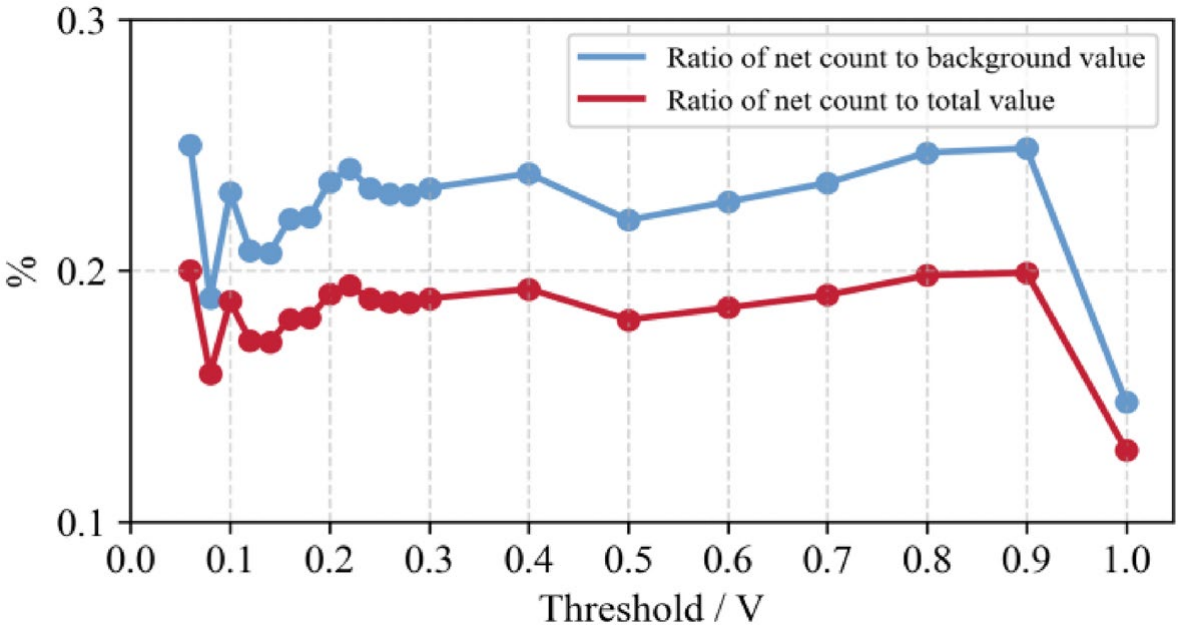
Ratio of net count to background value.

In order to determine the obvious threshold area for detection, the net count and background value of gangue placed under the same threshold are divided to obtain the counting increase of gangue placed under different threshold conditions, as shown in Fig. 10.

As depicted in Fig. 10 that before the threshold value 0.2 V, the counting amplitude fluctuates greatly. Between 0.2 and 0.9 V, the counting amplitude increases steadily and to a certain extent. At the threshold value of 1 V, the amplitude decreases significantly. Therefore, under the underground environment, the threshold value can be set between 0.8 and 0.9 V, for effective detection efficiency.

Based on the formed identification method of coal-gangue-rock in LTCC of extra thick coal seams, and taking the LTCC working faces of Lilou Coal Mine (thick coal seam with simple structure), Xiaoyu Coal Mine (thick coal seam with single layer dirt band) and Tashan Coal Mine (thick coal seam with complex structure) as specific conditions, the on-site installation scheme of detectors is designed to test the sensitivity, signal stability and adaptability to the environment of detectors; The response characteristics of the detector to the dirt band and roof rock are analyzed to provide a basis for determining the identification parameters.

**Figure 11:**
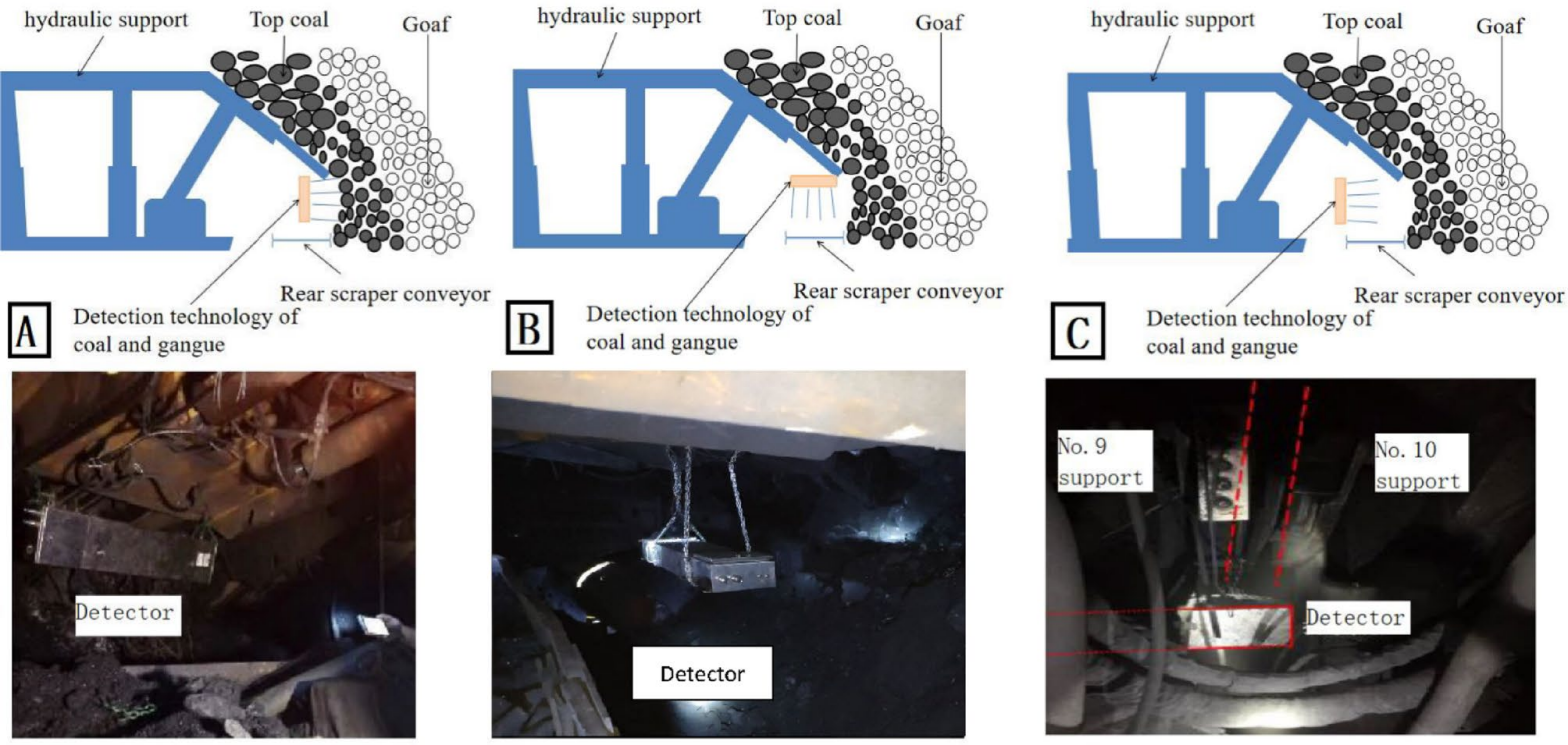
(**A**) Detector is installed under the support tail beam with the detection direction facing the coal chute. (**B**) Detector is installed above the rear scraper conveyor with the detection direction facing the coal flow. (**C**) Detector is installed under the support tail beam with the detection direction facing the coal chute.

KZT12 intrinsically safe coal gangue identification detector for mining has three installation positions: under the support tail beam with the detection direction facing the coal scupper, above the rear scraper conveyor with the detection direction facing the coal flow, and under the support tail beam with the detection direction facing the coal scupper, as shown in Fig. 11A–C.

## Result

### Application of coal gangue identification system in simple structure thick coal seam fully mechanized caving face

Working face 1303 (coal seam 3) of Lilou Coal Mine is located in the middle and lower part of Shanxi Formation. Most of the coal seams (coal seam 3) are relatively stable with simple structure. The average thickness of the coal seam is 6.98 m, the mining height is 3.6 m, the drawing height is 3.38 m, and the average dip angle of the coal seam is 13°. The immediate roof of the coal seam is sandy mudstone with a thickness of 0.96 m, and the basic top is fine sandstone with a thickness of 15.12 m.

KZT12 intrinsically safe coal gangue identification detector is installed under the tail beam of the support, with the detection direction facing the coal chute, as shown in Fig. 10A.

**Figure 12:**
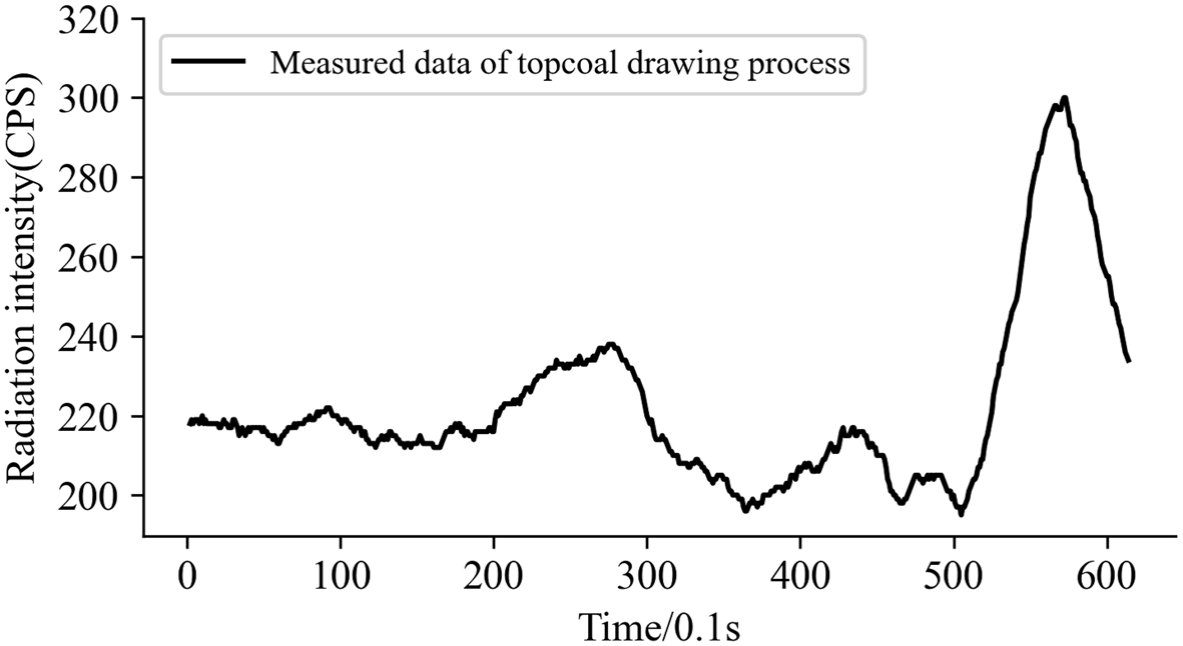
Radiation curve of top coal drawing process.

The radiation data and filtering data (Kalman filtering method) of coal gangue monitored during coal drawing are shown in Fig. 12.

As depicted in Fig. 12 that during top coal drawing, the detector has a relatively sensitive response to the occurrence and content of gangue. The detected radiation data has obvious periodicity, which can be divided into two stages: stage of pure coal and mixing stage of top coal and gangue. In the pure coal stage, there is pure coal near the caving-opening. Since there are almost no radioactive nuclides in the coal, the radiation curve of the radiation data detected by the detector fluctuates near the background value, but the fluctuation range is small. As the top coal being exhausted, the immediate roof rock gradually mixes into the caving-opening. At this stage, the radiation intensity increases significantly, indicating that a large amount of gangue is mixed,, the caving-opening operation is terminated in combination with the basis of “close the caving-opening when see the gangue”, The radiation curve gradually decreases and returns to the background level with the closing of the caving-opening.

### Application test of coal gangue identification system in LTCC face with single layer thick coal seam of dirt band

The coal seam of Working Face 8202 in Xiaoyu Coal Mine has a stable occurrence with little change. The thickness of the coal seam is 9.2–10.2 m, with an average thickness of 9.7 m. The top coal contains a layer of dirt band. The coal cutting height of the shearer is 3.2 m, the coal drawing height is 6.5 m, and the mining drawing ratio is 1:2.03.

KZT12 intrinsically safe coal gangue identification detector is installed above the rear scraper conveyor, with the detection direction facing the coal flow, as shown in Fig. 10B.

**Figure 13:**
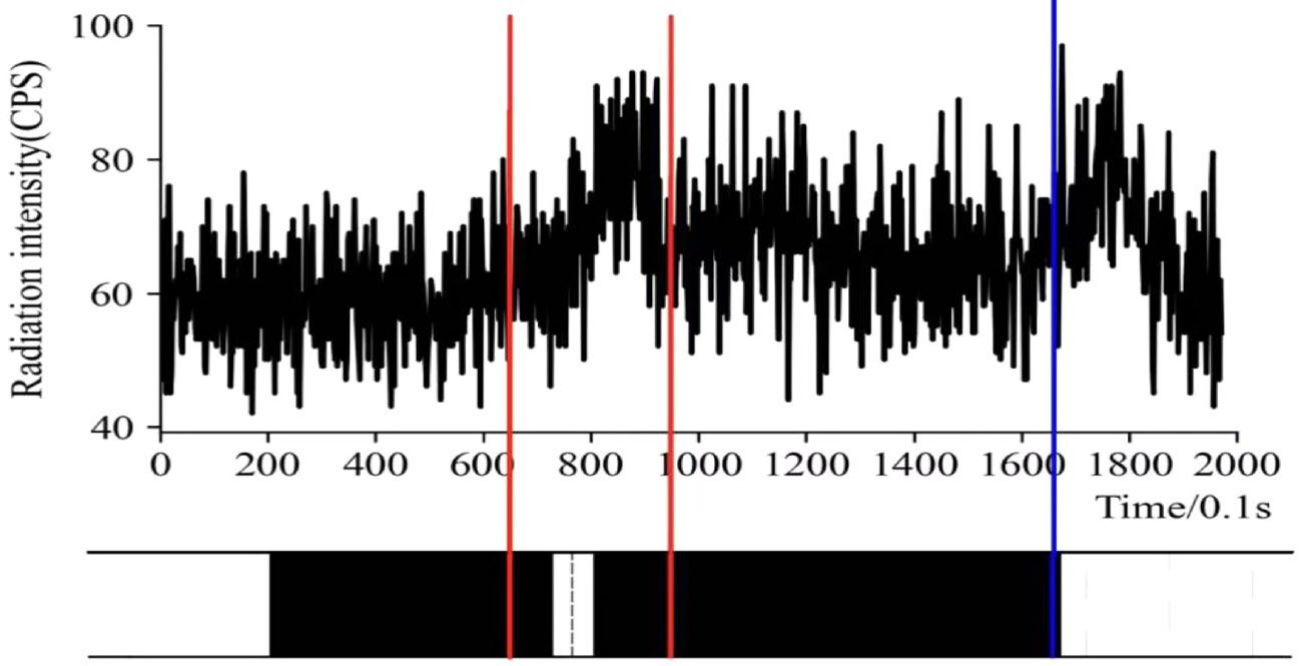
Radiation curve of top coal drawing process and its comparison with bar chart.

The radiation data and filtering data (Kalman filtering method) of coal gangue monitored during coal drawing are shown in Fig. 13. For the convenience of analysis, the occurrence of dirt bands in the top coal is compared with the data detected at the caving-opening.

As depicted in Fig. 13 that during top coal drawing, the detector has a relatively sensitive response to the occurrence and content of dirt band/roof rock. The process of top coal drawing lasts for 200 s, and there is a radiation wave peak. The radiation data detected has obvious periodicity, which can be divided into three stages: pure coal stage, gangue mixing stage and immediate roof rock mixing stage. In the pure coal stage, there is pure coal near the caving-opening. Since there is almost no radionuclide in the coal, the radiation data detected by the detector is at the same level with the background, that is, the radiation intensity fluctuates within 42–78 cps within 0–70 s after the caving-opening is opened, and there is no obvious upward or downward trend.

With the process of top coal drawing, the gangue containing in the top coal will reach the coal drawing opening. At this stage, the data detected by the detector will show an upward trend. With the full discharge of the gangue, the data detected by the detector will gradually decline, that is, in 70–95 s, the radiation intensity will first rise from 55 to 98cps, and then gradually decline; In the following 95–160 s, the coal drawing is a pure coal stage, and the radiation intensity is relatively stable; As the top coal is exhausted, after 160 s, the immediate roof rock starts to enter the coal drawing opening. At this stage, combined with the coal drawing basis of “see the gangue then close the caving outlet”, the coal drawing operation is terminated. The radiation curve gradually decreases and returns to the background level as the coal drawing opening is closed.

### Application test of gangue identification system in thick seam with complex structure

The average thickness of 8205 LTCC coal seam in Tashan Coal Mine is 15.09 m, the mining height is 3.8 m, the caving height is 11.29 m, and the mining caving ratio is 1:2.97. The cycle progress is 0.8 m, and the coal drawing step is 0.8 m. The coal seam contains 4–8 layers of dirt bands, with an average of 6 layers. The thickness of a single layer varies from 0.23 to 0.65 m. The lithology of the dirt bands is magmatic rock, sandy mudstone, mudstone, carbonaceous mudstone, and kaolinite. Most of the upper part of the coal seam is metamorphosed and silicified due to lamprophyre intrusion.

KZT12 intrinsically safe coal gangue identification detector is installed under the tail beam of the support, with the detection direction facing the coal chute, as shown in Fig. 10C.

**Figure 14:**
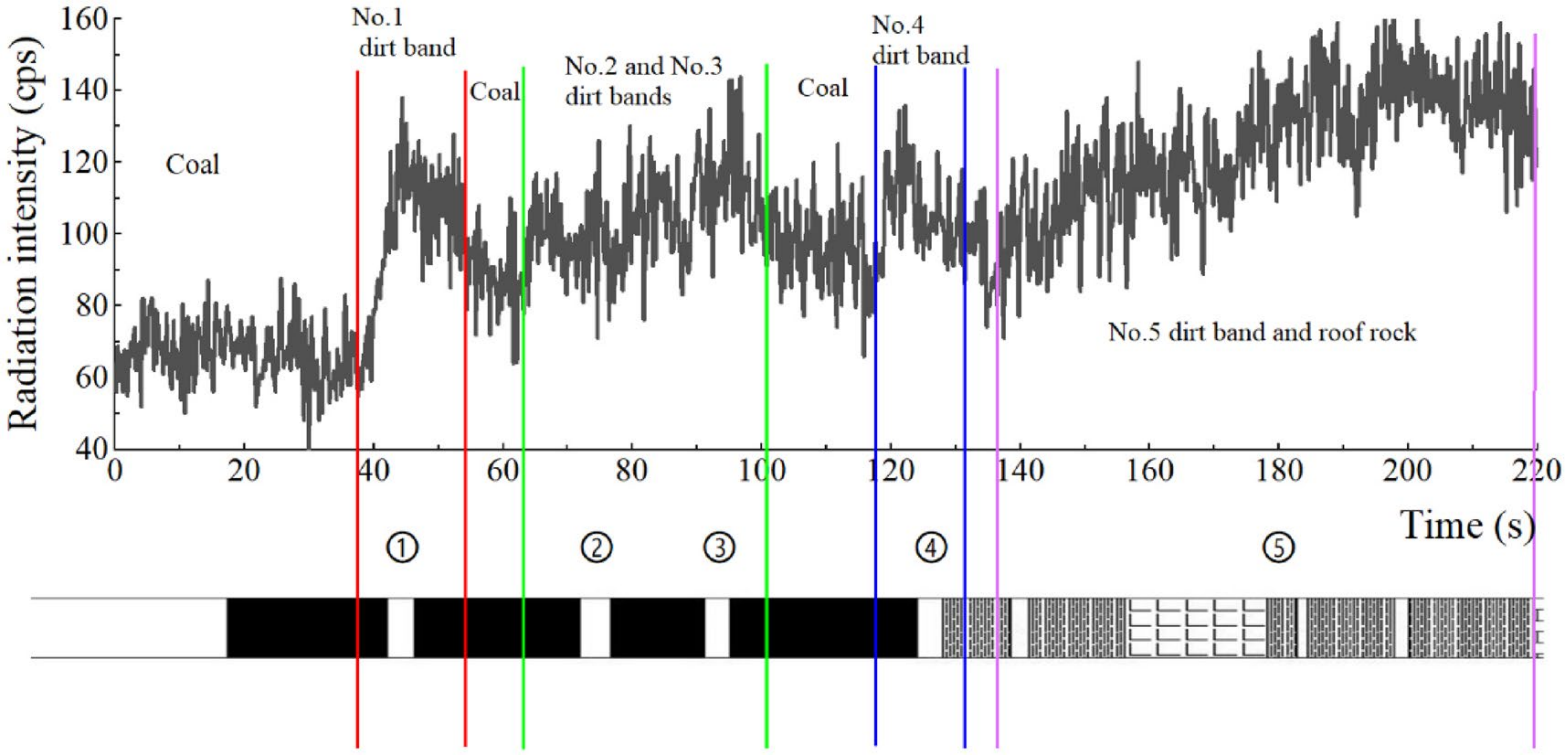
Radiation curve of top-coal drawing process and its comparison with bar chart.

The radiation data and filtering data (Kalman filtering method) of coal gangue monitored during coal drawing are shown in Fig. 14. For the convenience of analysis, the occurrence of dirt bands in the top coal is compared with the data detected at the coal drawing hole.

As depicted in Fig. 14 that during coal drawing, the detector has a relatively sensitive response to the occurrence and content of dirt band/roof gangue. The process of coal drawing at the caving-opening lasts for 220 s, and there are four radiation peaks. Due to the existence of dirt bands, the radiation data detected has obvious periodicity, which can be divided into three stages: pure coal stage, dirt band mixing stage and immediate roof mixing stage. The dirt band mixing stage can be subdivided according to the dirt band position. At the same time, the radiation performance of different lithology is different. The numbers of ① in the figure are the numbers of the dirt bands in the top coal, which are sorted from bottom to top. In the pure coal stage, there is pure coal near the coal cave outlet. Since there is almost no radionuclide in the coal, the radiation data detected by the detector at this stage is equal to the background, that is, the radiation intensity fluctuates within the range of 40–87 cps within 0–35 s after the coal cave outlet is opened, and there is no obvious upward or downward trend.

As the top coal caving process enters the mixed stage of coal and gangue, and the dirt band in the 1st layer of top coal reaches the coal caving outlet first. At this time, the data detected by the detector shows an upward trend. With the exhaustion of the 1st layer of dirt band, the data detected by the detector gradually decreases. That is, the radiation intensity first increases from 55 to 138 CPS and then gradually decreases within 35–58 s. Then the dirt band in layer ② arrives at the coal caving outlet, and the data detected by the detector shows an upward trend again. Because the distance between the dirt band layer ② and the dirt band layer ③ is relatively close, the time periods for these two layers to enter the top-coal caving-opening. Therefore, there is no obvious feature in the entry sequence on the radiation intensity curve, that is, for the 63–107 s period, the radiation intensity curve first slowly rises from 71 to 143 cps during the 63–95 s period,, and then decreases from 143 to 79 cps during the 95–107 s period, It can be found that the main reason for the long duration of the rising section during the mixing of the whole dirt band layer ② and the dirt band layer ③ is that the mixing amount of the dirt band layer ① is gradually decreasing, and the mixing amount of the dirt band layer ③ is gradually increasing as the mixing amount of the dirt band layer ② is gradually decreasing; The main reason for the short duration in the descending section is that the dirt band layers ①, ② and ③ are decreasing, and even the dirt band layers ① is no longer discharged through the coal caving outlet; At about 110s, the dirt band ④ is mixed in. During 110–137 s, during 110–115, the radiation intensity fluctuates within the range of 85cps to 107cps without obvious upward or downward trend, It is analyzed that at this time, the mixing amount of the dirt band layer ② and ③ decreases gradually, while the mixing amount of the dirt band layer ④ increases gradually, resulting in no obvious upward or downward trend of the radiation intensity; During 115–122 s, the radiation intensity curve rises from 66 to 134 cps. The analysis is that at this time, the mixing amount of the dirt band ④ increases to the maximum, while the mixing amount of the dirt band ② and ③ is very small; Then, during 122–138 s, the radiation intensity curve decreased from 136 to 71 cps, which was analyzed as a result of the gradual decrease in the mixing amount of the dirt band ④.

From 140 s to the end of 220 s coal drawing, the radiation intensity curve shows a slow upward trend. According to the comprehensive histogram of the working face and the histogram obtained by drilling before the experiment, The coal in the range from the dirt band layer ④ in the top coal to the top plate is mostly metamorphosed and silicified due to the intrusion of lamprophyre. This part of coal seam is defined as the dirt band layer ⑤, and this coal drawing stage is defined as the mixing stage of lamprophyre/ immediate roof, Therefore, with the mixing of this part of coal seams, the radiation intensity curve has an obvious upward trend at this time, and the recovery value of this part of coal is already small, so coal drawing will stop.

In the mixing stage of the dirt bands, due to the different location, thickness and lithology of the dirt bands in each layer, they show different radiation characteristics during the releasing process: ① The closer the position of the dirt band is to the caving-opening, the earlier the time of the caving-opening, which follows the time sequence of the top caving-opening; ② When the thickness of the dirt band is relatively large, the radiation monitoring curve corresponding to the mixing of the dirt band in the process of releasing is obviously rising and then slowly falling after maintaining the intensity for a certain time, That is, the dirt band is thick and will last for a period of time after being mixed into the coal drawing flow. When the dirt band is thin, the radiation monitoring curve corresponding to it shows a fluctuation phenomenon of one or more sections rising and then falling immediately from the mixing of the dirt band to the end during the releasing process, and the wave crest height of each section of the fluctuation has a certain difference, That is, the gangue layer is thin, which can not be continuously mixed into the coal drawing flow, and there is intermittent phenomenon, that is, the influence of different thickness of gangue on the radiation monitoring data is different, that is, the peak width of the monitoring curve is different; ③ The radiation intensity of the gangue with different lithology is different, so the influence on the radiation monitoring data is different during the mixing process. That is, the peak height of the monitoring curve is different. ④Two layers of dirt bands near each other will be mixed and overlapped.

In conclusion, the KZT12 intrinsically safe coal gangue identification detector has a high sensitivity to the amount of gangue discharged at the coal caving outlet, and can determine the gangue mixing ratio at the top coal caving process in real time.

## Discussion

LTCC mining technology is one of the main technologies for safe and efficient mining of extra-thick coal seams in China. At present, LTCC mining technology has been applied in most of the extra-thick coal seams in China, and has made breakthroughs and development in theory and key engineering application. However, the top-coal caving process in Full-mechanized top-coal caving still relies on manual control according to the principle of " close caveing-opening when see gangue", which is an artificial process. It is difficult to avoid the situation that resources are wasted and the quality of coal is affected due to over-or less-drowning in the process of top-coal caving. Moreover, the number of top-coal caving supports in the fully-mechanized top-coal caving face is large, the working environment of the top-coal caving procedure is poor, and the labor intensity and working efficiency of the manual control of the top-coal caving-opening are high.

In recent years, the intellectualization of coal mines has been developed rapidly. Taking intelligent fully mechanized mining as the technical core, it has improved the intelligent level of coal mines and provided technical support for the high-quality development of the coal industry. The development of intelligent fully mechanized mining technology has promoted the research on intelligent technology of LTCC. Because coal gangue identification is the key technology to realize automatic top coal caving and LTCC intelligent mining, domestic experts, scholars and scientific research institutions have carried out a lot of research work for this purpose and made gratifying progress. However, the instability of coal seam thickness and the existence of dust, dust falling water mist, brightness, space noise and other complex environments in the coal drawing space have brought great difficulties to the accuracy and reliability of coal gangue identification, which is also the reason why continue research have been carried out. For more than 10 years, the has successively carried out the application of near-infrared ray, dual energy γ Ray and nature γ based on the analysis and comparison of its reliability and feasibility, the coal gangue recognition based on low level radiation γ ray is proposed. The method of ray coal gangue recognition has been studied theoretically and experimentally, and tested and analyzed on the spot in Lilou Coal Mine, Tashan Coal Mine and Xiaoyu Coal Mine. The preliminary application shows that the proposed method and equipment for identifying coal gangue based on natural gamma ray can fully meet the requirements of real-time monitoring of coal gangue mixing degree at the top coal drawing process. Of course, the prerequisite for the application of coal gangue recognition method based on low level radiation γ ray is that the immediate roof rock needs to contain certain radioactive elements. According to the research in section ‘Radiation characteristics of coal and rock strata in typical thick coal seam mining areas in China’ of this paper, it has been proved that radioactive elements exist in the immediate roof of most thick coal seams in China.

Based on the complex conditions of the top coal caving opening in the LTCC mining face, in order to fully apply this technology to the top coal caving face, the author believes that the later research should focus on the following two points: (1) Installation position of coal gangue identification detector. Because of the different positions of detectors, first of all, the detection range of the detectors to the coal drawing mouth is different, and because of the influence of the floor and the gangue in the goaf, the noise source of the detectors also needs to be analyzed. Second, the monitoring range of the top coal is different due to the different positions of the detectors, which relates to the number of detectors installed and the way they cooperate with the electro-hydraulic control system. (2) The shape and size of the detector. In view of the complexity of the working environment of the coal chute and the space limitation, in order to achieve a good detection effect, in addition to the installation position, the size of the detector should also meet the detection requirements as far as possible. At the same time, in order to meet the safety requirements, in combination with the special structure of the hydraulic support, it is necessary to customize the detector with a special shape suitable for the coal chute space, which brings research challenges to the design and fabrication of detectors and the acquisition of signals. The team has carried out research on the above two points and believes that in the near future, better results will be displayed.

## Conclusions

1. Among the common gangue and roof rocks, the radioactive intensity of carbonaceous mudstone is the highest, followed by sandy mudstone and kaolin mudstone, while the radioactive intensity of lamprophyre is relatively minimum. When the gangue and the immediate roof lithology are the same, the radioactive intensity is similar. Coal has the lowest radioactivity. Through the analysis of the radiation intensity of the coal seam and roof in the typical thick seam LTCC mining face, it is feasible to use the natural γ ray method to identify the coal and gangue.
2. Based on the radiation characteristics of natural γ rays in the process of releasing coal and gangue in extra thick coal seams and the change law of radiation intensity value when the immediate roof is mixed γ value of radiation intensity is taken as the identification parameter, and the identification method of coal and gangue in LTCC mining of extra thick coal seam is proposed.
3. The KZT12 intrinsically safe coal gangue identification detector developed for mining has the ability to monitor the different mixing degrees of coal and gangue mixture in real time, and has good applicability to the identification of coal and gangue in the process of fully mechanized caving mining of extremely thick coal seams with complex structures. Because of nature γ ray has a strong penetrability, the detector is less affected by water mist, dust, light, etc. during mining, and has strong environmental adaptability, and can realize volume monitoring.
4. The automatic recognition method and system of coal and gangue in extra thick coal seams have been formed. The field test and analysis of coal gangue recognition have been carried out in the fully mechanized caving mining working faces of Lilou Coal Mine, Xiaoyu Coal Mine and Tashan Coal Mine, and remarkable technical effects have been achieved, laying a foundation for further research and application of intelligent fully mechanized caving mining in extra thick coal seams under different conditions.

## Data Availability

The original contributions presented in the study are included in this article, further inquiries can be directed to the corresponding author.
